# Accounting for heteroscedasticity and censoring in chromosome partitioning analyses

**DOI:** 10.1002/evl3.88

**Published:** 2018-11-13

**Authors:** Petri Kemppainen, Arild Husby

**Affiliations:** ^1^ Organismal and Evolutionary Biology Research Programme University of Helsinki 00014 Helsinki Finland; ^2^ Department of Ecology and Genetics Uppsala University 75236 Uppsala Sweden

**Keywords:** Chromosome partitioning, heritability, infinitesimal model, genomic relatedness, GCTA, SNP heritability

## Abstract

A fundamental assumption in quantitative genetics is that traits are controlled by many loci of small effect. Using genomic data, this assumption can be tested using chromosome partitioning analyses, where the proportion of genetic variance for a trait explained by each chromosome (*h^2^_c_*), is regressed on its size. However, as *h^2^_c_*‐estimates are necessarily positive (censoring) and the variance increases with chromosome size (heteroscedasticity), two fundamental assumptions of ordinary least squares (OLS) regression are violated. Using simulated and empirical data we demonstrate that these violations lead to incorrect inference of genetic architecture. The degree of bias depends mainly on the number of chromosomes and their size distribution and is therefore specific to the species; using published data across many different species we estimate that not accounting for this effect overall resulted in 28% false positives. We introduce a new and computationally efficient resampling method that corrects for inflation caused by heteroscedasticity and censoring and that works under a large range of dataset sizes and genetic architectures in empirical datasets. Our new method substantially improves the robustness of inferences from chromosome partitioning analyses.

**Impact summary**

Chromosome partitioning analyses, where the proportion of genetic variance for a trait explained by each chromosome (*h^2^_c_*) is regressed on its size, is a common way to test for a polygenic basis of traits. However, *h^2^_c_*‐estimates are censored to be positive and the variance in *h^2^_c_* increase with chromosome size, violating two assumptions of least squares regression. Using simulated and empirical data we demonstrate that these violations lead to incorrect inference of genetic architecture that depends on the number and size distribution of chromosomes, with 28% of published results being false positives. We introduce a new and computationally efficient resampling method that provides unbiased estimates and substantially improves the robustness of inferences from chromosome partitioning analyses.

Genome wide association studies (GWAS) in humans (Donnelly 2008; Yang et al. 2013; Timpson et al. 2018), livestock (Sharma et al. 2015) and natural plant and animal populations (Schielzeth and Husby 2014) have demonstrated that a wide variety of traits are controlled by many loci of individually small effect (Mackay et al. 2009), consistent with the infinitesimal model of quantitative genetics (Fisher 1930). When traits are polygenic, SNPs that reach statistical significance at a genome‐wide level typically only account for a small amount of the total narrow‐sense heritability (*h^2^*). This has fueled many discussions of “missing” or “hidden” heritability in GWAS studies (Manolio et al. 2009; Eichler et al. 2010; Yang et al. 2013).

One possible solution to the “missing heritability” problem is to consider the effect of all SNPs jointly, which should provide an unbiased estimate of the variance explained by all SNPs in the dataset, the so called SNP‐based heritability (*h^2^*_SNP_; Yang et al. 2010). For instance, *h^2^*_SNP_ was estimated to 45% for human height, compared to 1–3% when only considering genome wide significant SNPs (Yang et al. 2010). The SNP‐based heritability relies on causal variants being in linkage disequilibrium (LD) with genotyped SNPs and is a useful parameter also because it can be further partitioned among arbitrary portions of the genome, for example among intergenic and genic regions (Yang et al. 2010; Gusev et al. 2013; Yang et al. 2014; Loh et al. 2015). In particular, partitioning genetic variance among chromosomes (*h^2^_c_*) has proven a useful and popular approach to test for a polygenic basis of trait inheritance (Davies et al. 2011; Yang et al. 2011b; Jensen et al. 2012; Lee et al. 2012; Lee et al. 2013; Santure et al. 2013; Robinson et al. 2013; Yang et al. 2014; Berenos et al. 2015; Santure et al. 2015; Wenzel et al. 2015; Silva et al. 2017). If a trait is polygenic, then larger chromosomes (on average harboring more causal loci) are expected to explain more of the total *h^2^*_SNP_ and *h^2^_c_* is expected to scale positively with chromosome size (Yang et al. 2011b). Indeed, many human studies (Davies et al. 2011; Yang et al. 2011b; Lee et al. 2012, 2013; Yang et al. 2013, 2014) as well as studies on natural populations (Santure et al. 2013, 2015; Robinson et al. 2013; Berenos et al. 2015; Wenzel et al. 2015; Silva et al. 2017) have found significant regressions between *h^2^_c_* and chromosome size across a variety of different traits, suggesting that most traits are polygenic.

Chromosome partitioning tests are typically performed using ordinary least squares (OLS) regressions (Davies et al. 2011; Yang et al. 2011b; Jensen et al. 2012; Lee et al. 2012; Lee et al. 2013; Santure et al. 2013; Yang et al. 2013; Robinson et al. 2013; Yang et al. 2014; Berenos et al. 2015; Santure et al. 2015; Wenzel et al. 2015; Duan et al. 2016; Silva et al. 2017). However, standard errors of *h^2^_c_* estimates (*SE_h_*) increase with the number of SNPs (Visscher et al. 2014). This violates the assumption of homoscedasticity in OLS regression, something that can lead to bias in both the slope of the regression line (β) and the associated *P* value (Strutz 2016). Using simulated data we show that heteroscedasticity in combination with the fact that *h^2^_c_*‐estimates are constrained to be positive (censoring) leads to considerable *P* value inflation in chromosome partitioning analyses that use OLS regressions between *h^2^_c_* and chromosome size, something that can result in misleading inferences about the genetic architecture of traits.

One potential solution to mitigate *P* value inflation is to generate a null‐distribution by removing associations between genotype and phenotype prior to chromosome partitioning (by permutation). However, this is computationally demanding and complicated by the presence of population stratification that may add additional biases (Abney 2015). We use simulated and previously published empirical data from humans and other organisms and demonstrate that both heteroscedasticity and censoring in OLS regression between *h^2^_c_* and chromosome size can be accounted for by a simple and computationally efficient resampling procedure that is ideal for large genomic datasets. We demonstrate that our resampling procedure is robust to variation in genome characteristics as well as variation in the underlying genetic architecture of the trait, population stratification, and dataset size.

## Methods

### *P* VALUE INFLATION IN OLS REGRESSIONS BETWEEN h^2^_c_ AND CHROMOSOME SIZE IN DATA SETS SIMULATED UNDER THE NULL HYPOTHESIS

**Figure 1:**
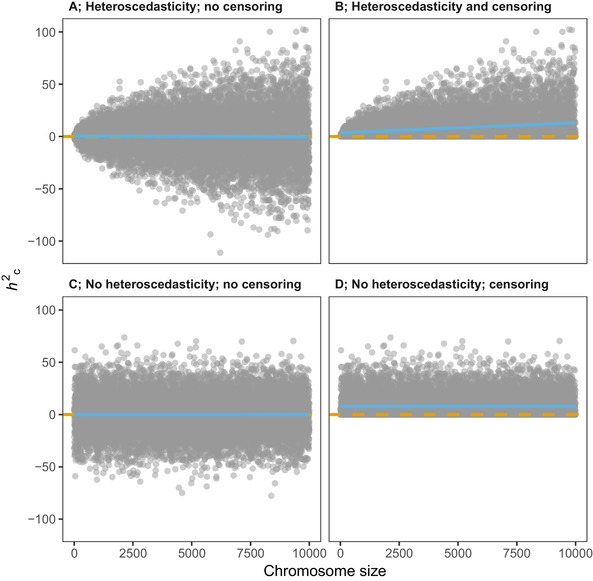
The expected effects of heteroscedasticity and censoring on OLS regression between *h^2^_c_* and chromosome size under the null hypothesis of no heritability. Simulated data for figures were produced by generating 10 k *x* values (representing chromosome size in arbitrary units, *z*, range: 1–10,000), and sampling *y* values (representing *h^2^_c_*) from a normal distribution with mean = 0 and *sd = sqrt*(*z*) (**A**); *sd = sqrt*(*z*) with negative values censored to 1 × 10^−6^ (**B**); *sd = *20 and no censoring (**C**); *sd = *20 with negative values censored to 1 × 10^−6^ (**D**). Without heteroscedasticity (**C** and **D**) slopes (β) of OLS regression lines are expected to be unbiased. While on average β’s are expected to be zero in (**A**), but with large variance, a bias towards positive β’s is expected in (**B**). Dashed orange line represents β under the null hypothesis (*y*‐intersect = 0, β = 0) and blue line represents linear regression line for all data points in each panel.

Theoretically, *h^2^_c_* estimates must be larger than or equal to zero (since they represent proportion of variance explained) and thus negative *h^2^_c_* estimates are typically censored to a small positive value by software that are used to partition heritability among chromosomes (e.g., GCTA, Yang et al. 2011a). In addition, *SE_h_* in these analyses are expected to increases with chromosome size (Visscher et al. 2014). As demonstrated in Figure 1, while only heteroscedasticity (Fig. 1A) or only censoring (Fig. 1D) are not expected to bias the mean β for regression lines (under the null hypothesis), the combination of both can severely bias both β's and *P* values (Fig. 1D). Thus, in the context of chromosome partitioning analyses, when *SE_h_* increases with chromosome size, but *h^2^_c_* cannot be negative, mean *h^2^_c_* is expected to increase with chromosome size even when there is no genetic basis of the trait. To test to what extent this causes *P* value inflation we performed chromosome partitioning analyses on data simulated under the null‐hypothesis of no association between genotype and phenotype. Earlier we have also demonstrated that variation in chromosome sizes and the numbers of chromosomes influence the power of chromosome partitioning analyses (Kemppainen and Husby 2018). We therefore tested the potential effect of these parameters on *P* value inflation in chromosome partitioning analyses using the chicken genome as a contrast to the human genome. While the human genome (Lander et al. 2001) comprises 22 chromosomes ranging from 47 to 250 mega base pairs (Mb), the chicken genome consists of 38 chromosomes ranging from <0.1 Mb to 196 Mb, the majority of chromosomes being so called “micro‐chromosomes” (20 of the smallest chromosomes are less than 5% of the size of the largest chromosome; Groenen et al. 2000). We excluded ten of the smallest chromosomes (<1 Mb) from the chicken genome in our simulations as they rarely contained any (or only a few) of the SNPs in each dataset. Thus for the simulated human genome we included 22 autosome pairs and for the chicken genome simulation 28 autosome pairs.

Population genomic datasets were simulated using Fastsimcoal2, version 2.5.2.21 (Excoffier and Foll 2011; Excoffier et al. 2013) assuming sexually reproducing, nonselfing individuals with nonoverlapping generations. For all datasets the total genome size was set to 1 Mb (but with different chromosome numbers and sizes directly scaled to the genome in question), and mutation rate, *μ* (with no transition bias), was adjusted such that at least *n_l_* = 10,000 biallelic SNPs (MAF > 0.05) would be generated for each dataset. Recombination between adjacent sites per generation, *r*, was set to 10 *μ* regardless of chromosome size and was uniform across chromosomes. Individuals were sampled from a single panmictic population with effective population size, *N*_e_ = 5000. We generated one thousand population genomic datasets for each species with *n_i_* = 2000 individuals and *n_l_* = 10,000 loci with phenotypic values sampled from a standard normal distribution with no association to genotype.

We used the software GCTA v1.24 (Yang et al. 2011a) to compute the chromosome‐specific genetic relationship matrices (GRMs) for each chromosome and estimated *h^2^_c_* by fitting the GRMs of all chromosomes separately (–grm and –reml options in GCTA) in the model: $y\;=g_{C}\;+\varepsilon$, where $g_{C}$ is a vector of genetic effects attributable to each chromosome and $var\;\left(g_{C}\right)=A_{C}\;\delta_{C}^{2}$ where $A_{C}$ is the GRMs from the SNPs on each chromosome and $\delta_{C}^{2}$ is the per chromosome variance. The proportion of variance explained by each chromosome is defined as $h_{C}^{2}=\delta_{C}^{2}/\delta_{P}^{2}\;$, with $\delta_{P}^{2}$ being the total phenotypic variance.

Under a polygenic model, *h^2^_c_* is expected to scale positively and linearly with the number of genes, *n_g_*, tagged by the SNPs on each chromosome (i.e., SNPs in LD with causal variants of a gene; Yang et al. 2010, 2011b). Assuming gene content and recombination rate is uniform across the genome and SNPs are randomly distributed, chromosome size (in base pairs) is a good and often used proxy for *n_g_* in these analyses. In our simulated data, recombination rate was uniform across chromosomes, and thus, the linkage map distances directly scales with physical distance. Because of the higher variability of SNP numbers in small chromosomes and the limited number of SNPs in our simulated datasets, we here used the number of SNPs on each chromosome as the proxy for *n_g_*, rather than chromosome size per se (still referred to as chromosome size throughout this manuscript). Linear relationships between *h^2^_c_* and chromosome size was tested with standard one‐tailed OLS regressions testing for the null‐hypothesis that *β* ≤ 0.

Under the true null‐hypothesis of a test, *P* values are expected to be uniformly distributed between 0 and 1 (Clayton et al. 2005). Deviations from this uniform distribution were estimated as the slope of a linear regression line in a quantile–quantile (QQ) plot based on observed versus expected –log_10_ *P* values and is equivalent to *P* value inflation (λ; Clayton et al. 2005). The presence of heteroscedasticity in the 2000 simulated datasets above was tested by regressing the means of the standard errors (SE*_h_*) for the *h^2^_c_*‐estimates (obtained from GCTA) against the square root of the number of SNPs on each chromosome.

### CORRECTING FOR HETEROSCEDASTICITY AND CENSORING IN CHROMOSOME PARTITIONING ANALYSES

One potential way to control for heteroscedasticity in a regression analysis is to use weighted least squares (WLS) regression. However, this is not expected to control for *P* value inflation arising from heteroscedasticity in combination with censoring. Another potential way to control for *P* value inflation arising from both heteroscedasticity and censoring, is to generate a null distribution of *P* values using permutations that is randomly changing the phenotypic values among sampled individuals in each dataset to remove any association between phenotype and genotype. In our data simulated under the null hypothesis this is guaranteed to produce an unbiased test (i.e. uniformly distributed *P* values) as in the simulated data there was no association between phenotype and genotype to begin with. However, permutation tests are slow and in addition challenging to perform in the presence of population stratification (Abney 2015).

Instead, we here introduce a method where a null distribution of *P* values is generated by sampling *h^2^_c_* values from a normal distribution with mean equal to zero and standard deviation (*sd*) equal to *SE_h_* for all regression data points for a given dataset. These data points are then censored as per standard in GCTA, that is replacing all negative *h^2^_c_*‐estimates with 1 × 10^−6^. This produces resampled datasets with the same pattern of heteroscedasticity and censoring as in the original dataset without the need for permuting phenotypic values and reanalyzing the data. If heteroscedasticity and censoring are the only sources of *P* value inflation in OLS regressions between *h^2^_c_* and chromosome size, we expect such resampled data with heteroscedasticity and censoring to generate similar distributions of (inflated) *P* values and *β’s* as simulated data under the null hypothesis. To test this, we generated resampled data (with heteroscedasticity only and with both heteroscedasticity and censoring) from the 2000 datasets simulated under the null hypothesis (see above) and compared the resulting *P* values and *β* distributions using QQ plots. If resampled data with heteroscedasticity and censoring produces similar distributions of *P* values as the underlying datasets simulated under the null‐hypothesis, we also expect *P* value null distributions from such resampled data to produce uniformly distributed *P* values, thus producing an unbiased test. From here on we refer to this procedure to simultaneously account for Heteroscedasticity and Censoring by resampling as “HC‐correction.” We also tested to what extent WLS regressions could control *P* value inflation by using 1/(*SE_h_^2)* as the weigh when fitting a linear model to the simulated or resampled data. Lastly, we also expect *P* values from HC‐correction and simple permutation of phenotypic values (see above) to be highly correlated, not only under the null hypothesis, but also when null hypothesis is not true (i.e., *h^2^* > 0). To test this, 100 population genomic datasets were simulated with *n_i_* = 1000 and *n_l_* = 5000 for both chicken and human genomes with *h^2^ = *0 or *h^2^ = *0.5. The smaller number of samples and loci were used to limit computational time for the permuted datasets, otherwise simulation parameters were as described above. For data sets with *h^2^ = *0, phenotypic values were generated as above. When *h^2^ = *0.5, a polygenic architecture was simulated by randomly sampling 100 causal loci from each dataset (i.e., all phenotypic variance was captured by our causal loci). Phenotypes were then simulated based on the causal loci (following documentation to GCTA software, version 1.24; Yang et al. 2011a) assuming an additive genetic model $y_{ij}=\sum_{i}w_{ij}\;\times u_{i}+\varepsilon_{j}$, where $w_{ij}=\left(x_{ij}-2p_{i}\right)/\sqrt{2p_{i}\left(1-p_{i}\right)}\;$, *x_ij_ is* the number of reference alleles for the *i*‐th causal variant of the *j*‐th individual, *p_i_* is the frequency of the *i*‐th causal variant, *u_i_* is the allelic effect size of the *i*‐th causal variant and *e_j_* is the residual effect. The allelic effect sizes were sampled from a standard normal distribution. The residual effect was generated from a normal distribution with mean of 0 and variance equal to $var\left(\sum_{i}w_{ij}\times u_{i}\right)/\left(1-1/h^{2}\right),$ where the narrow sense heritability, *h^2^ = V*_A_/*V*_P,_ *V*_A_ being the additive genetic variance and *V*_P_ the total phenotypic variance.

Null distributions for observed *P* values were generated either by permuting phenotypic values among individuals in a simulated data set or by resampling *h^2^_c_* estimates with heteroscedasticity and censoring as described above. To avoid unnecessary resampling, we continued permutation or resampling adaptively (Che et al. 2014) until either the number of *P* values from OLS regressions between *h^2^_c_* and chromosome size were more significant than the observed *P* value (*a*), or the total resampling replicates were calculated with *R* total successes (*b*), where *R* < *a*. We set the precision level, $c\;=\;SE(\hat{P})/\alpha$ to 0.2 where type I error rate, α = 0.05, thus assuring $SE(\hat{P})$at α = 0.05 is less than *c*α = *0.01*. With these settings, following guidelines in Che et al. (2014), we continued resampling until either *a = *34 ($\hat{P}$ *= a*/*B*) or *b = *475 ($\hat{P}$ = (*R*+1)/(*b*+1)).

### EVALUATING HC‐CORRECTION USING EMPIRICAL DATA

If heteroscedasticity and censoring are the only factors determining *P* value inflation in chromosome partitioning analyses, we further expect chromosome partitioning to produce similar (but not necessarily identical) relationships between HC‐corrected and uncorrected *P* values in empirical and simulated data, given the chromosome number and size distribution (as used in the chromosome partitioning analyses in the empirical data) are exactly the same. To test this, and to evaluate the presence of *P* value inflation in chromosome partitioning analyses in empirical data more generally, we reviewed the literature. In order to compare the empirical data to simulated data we generated data under a broad spectrum of dataset sizes and genetic architectures (where *h^2^* > 0; see below), but always exactly matching the chromosome size distribution in the corresponding empirical data set. Based on our literature review, the five empirical datasets presented in Table 1 were the largest available with respect to number of traits for the same dataset, and where information about chromosome sizes as well as both *h^2^_c_* and *SE_h_* for each of the chromosome partitioning analyses (the data necessary for HC‐correction) were available. For all datasets except humans (see below) we used the number of SNPs for each chromosome as a proxy for chromosome size in the matching simulated datasets, as for some datasets (collared flycatchers and great tits), SNPs were not randomly sampled from the genome. To achieve convergence in chromosome partitioning analyses in some of the published studies on empirical bird datasets, loci from the micro‐chromosomes were either pooled (great tits) or successively removed (starting from the smallest chromosome, until convergence was achieved; collared flycatchers and house sparrows). When chromosomes were removed to achieve convergence, we only used the chromosomes that converged for all phenotypic traits for a given genomic dataset to maximize the number of data points with exactly the same chromosome number and size distributions. Thus, our results with uncorrected *P* values are not necessarily directly comparable with the original results. Since only information for chromosome size was available for human data set (number of SNPs and chromosome sizes are however highly correlated in this data; Yang et al. 2011b), we used chromosome sizes directly to determine size of the simulated human chromosomes, instead of the number of SNPs for each chromosome. Note that the final number of SNPs per chromosome were not necessarily the same in all simulated data sets (or compared to the empirical datasets) as the number of SNPs (for a given level of *n_l_*) was a random sample of all the polymorphic SNPs in the simulated data that passed the filtering criteria.

**Table 1:** Summary of empirical datasets

Dataset	*n_l_*	*n_i_*	Stratification	No. of traits *
Humans^1^	565,040	6641–11,578	Unrelated	5 (2/2)
Great tits^2^	5312	416–1949	Family structure	17 (10/2)
Soay sheep^3^	37,037	5805	Family structure	5 (2/1)
House sparrows^4^	6196	721–1448	Family structure	7 (3/1)
Collared flycatchers^4^	40,822	798—800	Family structure	4 (2/2)

*n_l_*, number of loci; *n_i_*, number of individuals. ^*^Values in brackets indicate number of significant regressions between *h^2^_c_* and chromosome size, before/after HC‐correction (see also Table S1). References: ^1^Yang et al. 2011b, ^2^Santure et al. 2015, ^3^Berenos et al. 2015, ^4^Silva et al. 2017.

For all chromosome size distributions (matching a given empirical data set), we simulated 100 datasets for each combination of levels of the factors *n_i_* (1000 or 2000), *n_l_* (5000 or 10,000) with *h^2^ = *0.5, in total 400 datasets (with phenotypes generated as described above). In addition, for all these datasets, phenotypic values were permuted to generate 400 additional datasets with no association between phenotype and genotype (*h^2^ = *0). No attempt was made to match these parameters with the empirical data (except for the chromosome size distribution). This was both deliberate and practical as the main objective was to compare the results of chromosome partitioning analyses with matching genome characteristics, simulating complicated population structure (similar to what is observed in the empirical datasets) is challenging and simulating large datasets is computationally demanding. To estimate HC‐corrected *P* values we increased *b* to 49,9975 (instead of 475, see above) to ensure $SE(\hat{P})$would be less than *c*α *= 0.01* at α = 0.05/1000, all other parameters for the adaptive resampling being as defined above. To calculate HC‐corrected *P* values for empirical datasets we further increased *a* to 120 and b to 49,9975 to ensure that $SE(\hat{P})$ is no lower than *c*α *= 5 × 10^−3^* at α = 0.05/1000 (with other parameters kept as above), thus ensuring higher precision for HC‐corrected *P* values for the empirical datasets compared to the simulated datasets.

We tested whether the HC‐correction in the empirical data differed from their respective simulated data by first fitting a loess regression line in R (R Core Team 2015) to the simulated data and then using a paired *t*‐test to asses if there was a difference in the predicted HC‐corrected *P* values and the observed empirical HC‐corrected *P* values.

To test the effect of population structure on our HC‐correction approach we simulated 400 additional datasets with two populations (*N_e_* = 2500 each) connected by two migrants per generation in mutation drift balance, for human genomes (*n_i_* = 1000 or 2000; *n_l_* = 5000 or 10000 and *h^2^ = *0.5, as described above). We fitted the GRMs of all the chromosomes simultaneously (‐mgrm option in GCTA software) in the model $y\;=\sum_{C=1}^{n_{C}}g_{c}\;+\varepsilon$ where $n_{C}$ is the number of chromosomes for the analysis. Due to convergence limitations, datasets simulated with population structure were restricted to *h^2^ = *0.5 and human genomes (with population structure we could not perform analyses on data simulated under the null hypothesis of *h^2^ = *0, see Discussion).

## Results

### *P* VALUE INFLATION UNDER THE NULL HYPOTHESIS

**Figure 2:**
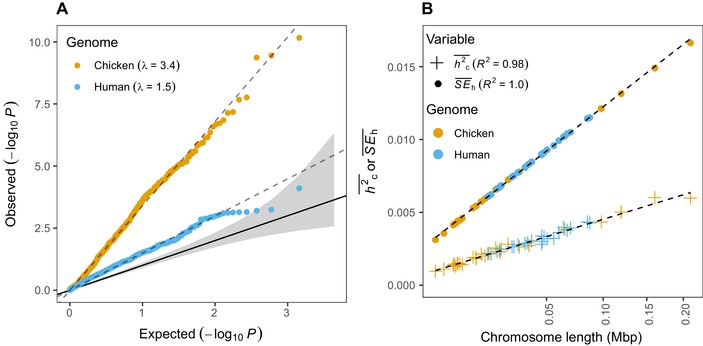
*P* value inflation in simulated data when there is no association between phenotype and genotype (*h^2^ = *0). (**A**) Shows QQ‐plot of expected (uniform distributions between 0 and 1) versus observed (‐log_10_) *P* values from OLS regression between *h^2^_c_* and chromosome size. *P* value inflation (λ) is the slope for the regression line (dashed). Gray area shows 95% confidence interval and solid black line indicates 1:1 line. (**B**) Shows the means of *SE_h_* and *h^2^_c_* (based on one thousand simulated datasets) for each chromosome as a function of chromosome size (square root scaled axis). Adjusted *R^2^* for regression lines (dashed) are indicated in figure. See Methods for additional simulation parameters.

In the chicken datasets simulated under the null hypothesis of no heritability, –log_10_ *P* values from one‐tailed OLS regressions between *h²_c_* and chromosome size were inflated by a factor of 3.4 and among the human datasets by a factor of 1.5 (Fig. 2A). Thus, the standard way in which chromosome partitioning analyses test for polygenic architecture is anti‐conservative and biased to infer a polygenic architecture. Indeed, to reach the true significance level the nominal *P* value would have had to be 3.8 × 10^−5^ and 0.011, leading to 42% and 14% false positives for chicken and human datasets, respectively (instead of the expected 5%, at false‐discovery rate α = 0.05).

In these data sets (with *h^2^ = *0) we found that mean *SE_h_* increases linearly with the square root of chromosome size (as measured by the number of base pairs; *P* < 0.001, β = 3.3 × 10^−5^, adjusted *R* ^2^ = 1.0; Fig. 1B), thus, heteroscedasticity is prevalent. Under the null hypothesis of no association between phenotype and genotype, the means of *h²_c_* for each chromosome ($\bar{h_{c}^{2}}$) should be centered around zero and thus the slope of the regression line, β, between $\bar{h_{c}^{2}}$ and chromosome size is not expected to deviate from zero. However, due to heteroscedasticity and the fact that *h²_c_‐*estimates are constrained to be positive by GCTA, the means of *h²_c_* are always positive and they also scale linearly with the square root of chromosome size (*P* < 0.001, β = 1.3 × 10^−5^, adjusted *R* ^2^ = 0.98; Fig. 2B). This inevitably causes *P* value inflation in regression tests between *h^2^_c_* and chromosome size, as seen in Figure 2A.

### CORRECTING FOR HETEROSCEDASTICITY AND CENSORING IN CHROMOSOME PARTITIONING ANALYSES

In datasets with both heteroscedasticity and censoring, WLS regression (that control for heteroscedasticity but not censoring) reduced *P* value inflation in both human and chicken datasets, but was not sufficient to remove it (Fig. S2). Particularly in chicken datasets substantial *P* value inflation remained (Fig. S1). One frequently used alternative to generate unbiased distribution of test statistics is permutation tests and as expected this produced uniformly distributed *P* values for both the simulated chicken and human genome datasets and resulted in unbiased tests (Fig. S2A).

**Figure 3:**
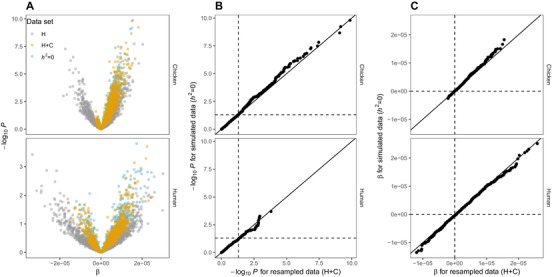
Simulated data compared to resampled data with heteroscedasticity and/or censoring. One thousand simulated datasets with *h^2^ = *0 (data as in Fig. 2) are compared to data where *h^2^_c_*‐estimates are resampled from a normal distribution (mean equal to zero and *sd* equal *SE_h_*), with (H+C) or without (H) censoring all negative values to 1 × 10^−6^. Shown are –log_10_ *P* values plotted against β from OLS regressions (two‐tailed) between *h^2^_c_* and chromosome size (A) and Q–Q plots comparing –log_10_ *P* values (B) and β (C) from the simulated data and the resampled data (H+C). Upper and lower panels for each figure show results from chicken and human datasets, respectively. Horizontal and vertical dashed lines in indicate significance level at α = 0.05 (B) and 0 (C). In unbiased tests, ∼5% of values under the null hypothesis are expected to significant, and β’s are expected to be centered on zero.

Next, we explore how heteroscedasticity alone and in combination with censoring bias β's and *P* values in chromosome partitioning analyses. In Figure 3, we compare datasets simulated with no heritability (*h^2^ = *0) with data resampled with heteroscedasticity and censoring (H+C) and heteroscedasticity without censoring (H). As expected (based on Fig. 1), with resampled data with heteroscedasticity but no censoring, β’s were centered around zero (Fig. 3A). With heteroscedasticity and censoring there was a bias toward positive values and this bias was stronger for chicken genomes compared to human genomes (Fig. 3A and B). In addition, the resampled data with heteorscedasticity and censoring are indistinguishable from the simulated data (when *h^2^ = *0) with respect to both β‘s and *P* value, for both chicken and human genomes (Fig. 3B,C). This is because $SE\;=sd/\sqrt{\left(n\right)}\;$ where *n* is the sample size and since *n* is a constant *sd* scales linearly with *SE* for a given dataset. This demonstrates that in our simulated data heteroscedasticity and censoring is enough to explain the observed *P* value inflation in regressions between *h^2^_c_* and chromosome size.

Because data resampled with heteroscedasticity and censoring produces equivalent distributions of *P* values as data generated under the null hypothesis, HC‐correction (for the two thousand datasets presented in Fig. 2A) produced uniformly distributed *P* values (Fig. S2B). More importantly, *P* values from permutation and HC‐correction were highly correlated (*R* ^2^ > 0.83; Fig. S3) both when the null hypothesis was correct (*h^2^ = *0) as well as when the true underlying genetic architecture was polygenic with *h^2^ = *0.5, demonstrating that these methods are comparable. There was an expected upward shift in the range of –log_10_ *P* values in data simulated under the null hypothesis compared to when the trait was polygenic (Fig. S3).

It took approximately 13,000 times longer (on a standard i5 8600 Intel core desktop computer using a single core) to generate null distributions by permutation (22 s/replicate), compared to HC‐correction (1.7 ms/replicate), for the above datasets. Moreover, HC‐correction is independent of size of the genomic dataset, in contrast to the permutation approach where time per replicate increased approximately sixfold when doubling of the number of individuals (but was not affected much by the number of loci in the dataset).

### HC‐CORRECTION IN PUBLISHED EMPIRICAL DATA

**Figure 4:**
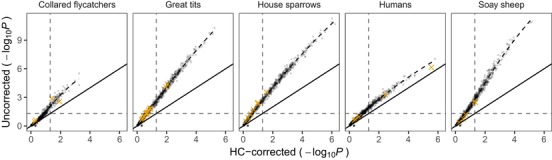
*P* values from chromosome partitioning in simulated and empirical data. Data were simulated both when the null hypothesis is true (*h^2^ = *0) and when the trait was polygenic (*h^2^ = *0.5). When there is no *P* value inflation, uncorrected *P* values should equal HC‐corrected *P* values (solid black line indicates 1:1). The slope of the loess regression line fitted on simulated data indicates magnitude of *P* value inflation for each dataset. Black dots represent simulated data and yellow crosses are empirical data points. Horizontal and vertical dashed lines indicate significance level at α = 0.05, such that data points in the upper left quadrant represent false positives, if not accounting for heteroscedasticity and censoring.

The resampling method (HC‐correction) is only useful if it also works for empirical data, which potentially have much more complex genetic architectures and population demographic history compared to simulated data. We have demonstrated that genome characteristics have a strong effect on *P* value inflation in simulated data (Fig. 2A). If this is the only factor determining *P* value inflation in chromosome partitioning analyses, we expect HC‐correction to produce similar results in empirical and simulated data, given the chromosome number and size distribution (as used in the chromosome partitioning analyses in the empirical data) are exactly the same. Figure 4 shows that the empirical data for each species follow their own (simulated) distribution much more closely than those from the other species. However, the empirical datasets differed significantly (paired *t*‐tests) with respect to the relationships between uncorrected and HC‐corrected *P* values in Soay sheep (*t_4_* = 3.7, *P = *0.02), great tits (*t_14_* = 3.3, *P = *0.006), and house sparrows (*t_6_* = 4.7, *P = *0.003), but not in humans (*t_4_* = –0.04, *P* > 0.05) or collared fly catchers (*t_3_* = 0.4, *P* > 0.05). The ratio between HC‐corrected and uncorrected *P* values (λ_cor_) can be viewed as a point estimate of *P* value inflation (otherwise *P* value inflation can only be estimated when a large number of tests have been performed; Kemppainen and Husby 2018). As seen in Figure S4 (where λ_cor_ is plotted against the uncorrected *P* value), the level of *P* value inflation that can be expected in chromosome partitioning analyses (when not accounting for heteroscedasticity and censoring) to some extent depends on the strength of the relationship between chromosome size and *h^2^_c_* (i.e., the effect size). This is also evident in Figure 4, where the relationship between HC‐corrected and uncorrected *P* values is not strictly linear. However also in Figure S4 the relationship between λ_cor_ and the uncorrected *P* value (used as a proxy for effect size), the difference between simulated and empirical data within each species is small relative to that observed between species.

The small differences between empirical and simulated datasets could be due to factors other than variation in chromosome number and size distribution, such as, for example, genome architecture and population stratification. Figure S5, shows that (moderate) population structure to some extent effects the relationship between HC‐corrected and uncorrected *P* values, but again, the difference between datasets with and without population structure is small relative to the effect of chromosome number and size distribution. In addition, among the 400 simulated datasets, there was no difference in the ratio between significant and nonsignificant HC corrected *P* values (χ^2^‐test; *P* > 0.05) between datasets with and without population structure (217 significant and 183 nonsignificant tests vs 229 significant and 171 nonsignificant for data sets with and without population structure, respectively).

Among the 36 empirical chromosome partitioning tests (Table 1 and Table S1), 18 were significant prior to HC‐correction and eight remained significant after HC‐correction, thus 28% of the tests were false positives due to not accounting for heteroscedasticity and censoring in the regression test between *h^2^_c_* and chromosome size.

## Discussion

Large‐scale genotyping studies can substantially contribute to the central goal of understanding and defining the genetic architecture of complex traits in humans (Timpson et al. 2018) and in evolutionary genetics more generally (Schielzeth and Husby 2014). Many studies use chromosome partitioning to test for a polygenic basis of traits by regressing chromosome specific heritability estimates on chromosome size (Yang et al. 2011b; Santure et al. 2013, 2015; Robinson et al. 2013; Berenos et al. 2015). However, we show here that heteroscedasticity in combination with censoring causes biased parameter estimates and *P* value inflation in chromosome partitioning analyses (Figs. 2, 3, 4), which can lead to overconfidence in a polygenic basis of traits. Lack of support for polygenic trait inheritance could, for example be due to low power (low number of loci or low *h^2^* of the trait), oligogenic trait inheritance, skewed effect size distributions, or that causal loci are not randomly distributed in the genome (Kemppainen and Husby 2018). Using simulated data we show that the magnitude of *P* value inflation depends on the number and size distribution of chromosomes of the species in question; *P* value inflation is much higher when number of chromosomes and variation in chromosome sizes are large, as is found in bird genomes (*λ* = 3.4) compared to human genomes (*λ* = 1.5; Fig. 2; see also Kemppainen and Husby 2018). Under the null hypothesis of no association between phenotype and genotype, not accounting for heteroscedasticity and censoring resulted in 42% and 14% false positives, for chicken and human datasets, respectively, instead of the expected 5% (at α = 0.05).

With simulated data, we further demonstrate that using null‐distributions for OLS regression *P* values from either *i*) permutation of phenotypic values prior to chromosome partitioning analyses, or (*ii*) resampling *h^2^*‐estimates from a normal distribution with mean equal to zero and *sd = SE_h_* with censoring (HC‐correction) accounts for the *P* value inflation under the null hypothesis (Fig. S2), and that *P* values from both approaches are highly correlated (Fig. S3). Thus, heteroscedasticity and censoring causes the observed *P* value inflation in the simulated data with *h^2^ = *0 and accounting for these biases with permutation or HC‐correction leads to comparable and unbiased tests.

However, permutation of phenotypic values has two major drawbacks: first, it is computationally demanding since separate chromosome partitioning analyses need to be performed on each permuted dataset. Second, and more importantly, in the presence of population stratification (as is present in virtually all empirical datasets), naively permuting phenotypic values among all individuals in a dataset can lead to an invalid test (Abney 2015). HC‐correction avoids these shortcomings by directly addressing biases caused by heteroscedasticity and censoring by resampling, and is therefore well suited for large empirical genomic data where computational speed is of concern.

The simulated data were generated under simple population genetic scenarios and genetic architectures, while empirical data potentially have much more complex population demographic histories, patterns of population stratification and genetic architectures of phenotypic traits, something that could potentially affect our HC‐correction approach. In addition, particularly in humans, the size of the simulated data substantially differed from the empirical data (5000 vs 565,040 loci and 1000 vs 11,578 individuals in simulated and empirical data, respectively; Table 1). Despite this, the relationship between uncorrected and HC‐corrected *P* values from data simulated under a variety of dataset sizes with polygenic trait inheritance were not substantially different from empirical data with matching chromosome numbers and size distributions (Fig. 4 and Fig. S4). However, there were some significant differences between the empirical and simulated data that were not fully accounted for by genome characteristics–this is apparent in the way that the empirical data in Figure 4 and Figure S4 do not perfectly match the simulated data. This could, for instance, be due to population stratification (as also shown for simulated data with population structure; Fig. S5) or other effects. Importantly, this does not necessarily imply that HC‐correction in empirical data is biased as we do not know the true distribution of *P* values in empirical data when the null‐hypothesis is not true and what/how other factors apart from population stratification and genome characteristics may affect it.

Due to convergence issues (see below) we could not evaluate *P* value inflation in datasets with population structure under the null hypothesis of *h^2^ = *0. Nevertheless, HC‐correction addresses the substantial part of the *P* value inflation (that can be explained by genome characteristics, and is caused by violating the assumptions of homoscedasticity and noncensoring of data) in empirical data, and doing so will, in future, make other, more minor effects, easier to address. How much population stratification, for example strong family structure also biases chromosome partitioning analyses, beyond what can be addressed by HC‐correction, remains also to be tested in the future.

In the software used for chromosome partitioning, GCTA (Yang et al. 2011a), *h²_c_* can be estimated for all chromosomes jointly (joint analyses, option ‐mgrm) or for each chromosome separately (separate analyses, option ‐grm). While the separate analyses are sensitive to population stratification in the data (otherwise joint and separate analyses produce equivalent results; Yang et al. 2011b), it is not possible to achieve convergence in the joint analyses when heritability for all chromosomes is zero (and is difficult also when the majority of chromosomes only explain small portions of the total phenotypic variance). Thus, in order to evaluate the possibility of *P* value inflation under the null hypothesis of *h^2^ = *0, the simulated populations were here assumed to be panmictic, such that no bias would be introduced when *h²_c_* for each chromosome were estimated separately. Although separate analysis almost certainly leads to biased results in empirical datasets and is therefore not recommended (except for the purpose of comparison; Yang et al. 2011b), the high similarity of the relationships between HC‐corrected and uncorrected *P* values in simulated and empirical data (Fig. 3; Fig. S4) in our analyses suggests that this is unlikely to have introduced any strong bias.

There is an option in GCTA (–reml‐no‐constrain) that allows negative estimates so that the mean from multiple replicates is unbiased. In theory, with this option it should be possible to correct for heteroscedasticity using WLS regression (as then there is no censoring). However, we experienced significant convergence issues with this option even for human genomes (where convergence was less problematic than in chicken genomes) and when overall heritability was high (0.5). While removing chromosomes with low heritability from the analyses would eventually lead to convergence, this would bias results towards large (nonnegative) *h^2−^*estimates similarly to censoring that also would have to be addressed to produce a nonbiased test. The “–reml‐no‐constrain” option in combination with WLS regression is therefore not of any practical use to address the *P* value inflation.

While many studies in human genetics have tested for a polygenic basis of traits using OLS regressions between *h^2^_c_* and chromosome size, we only found one where the necessary information for HC‐correction was publicly available. In contrast, we found several studies from natural populations that reported sufficient information to allow us to apply our HC‐correction (Table 1). As the information needed for HC‐correction is just chromosome size (or other proxy that is expected to correlate strongly with the number of genes per chromosome), *h²_c_* and *SE_h_*, this should be simple to report and we recommend all studies to do this in the future, also to facilitate possible meta‐analyses. If these parameters are reported, it is possible to reanalyze published data on chromosome partitioning to correct for the *P* value inflation. For instance, of the 36 different chromosome‐partitioning tests that we reanalyzed, only 8 out of 18 significant tests (using uncorrected *P* values from OLS regression) remained significant after HC‐correction (Table S1). This clearly demonstrates the need for HC‐correction in genomic studies aiming to understand the genetic architecture of traits, particularly in species with larger number of chromosomes and range in chromosome sizes where *P* value inflation is particularly prevalent.

## DATA ACCESSIBILITY

Code used to produce simulated data and analyses and raw data will be submitted to data dryad upon accepted manuscript.

## Supporting information


**Figure S1**. Ordinary least squares (OLS) regression versus weighted least squares regression (WLS) with heteroscedasticity and censoring.
**Figure S2**. Correction of *P* value inflation under the null hypothesis using permutation or resampling with heteroscedasticity and censoring.
**Figure S3**. *P* value correction using null distribution from permutation or resampling with heteroscedasticity and censoring.
**Figure S4**. The ratio between HC‐corrected *P* values and uncorrected *P* values (λ_cor_) depends on the strength of correlation between *h^2^_c_* and chromosome size.
**Figure S5**. Relationship between uncorrected and HC‐corrected *P* in simulated data with population structure.
**Table S1**. Uncorrected (OLS) and HC‐corrected (HC) *P* values from published chromosome partitioning analyses.
