# Supplementary material for: Accounting for heteroscedasticity and censoring in chromosome partitioning analyses
Source: Evol Lett. 2018 Nov 13;2(6):599–609. doi: 10.1002/evl3.88 (PMC6292708; doi:10.1002/evl3.88)
Supplement: Supplementary file 1 — Figure S1. Ordinary least squares (OLS) regression versus weighted least squares regression (WLS) with heteroscedasticity and censoring. Figure S2. Correction of P value inflation under the null hypothesis using permutation or resampling with heteroscedasticity and censoring. Figure S3. P value correction using null distribution from permutation or resampling with heteroscedasticity and censoring. Figure S4. The ratio between HC‐corrected P values and uncorrected P values (λcor) depends on the strength of correlation between h2c and chromosome size. Figure S5. Relationship between uncorrected and HC‐corrected P in simulated data with population structure. Table S1. Uncorrected (OLS) and HC‐corrected (HC) P values from published chromosome partitioning analyses. [file EVL3-2-599-s001.docx]

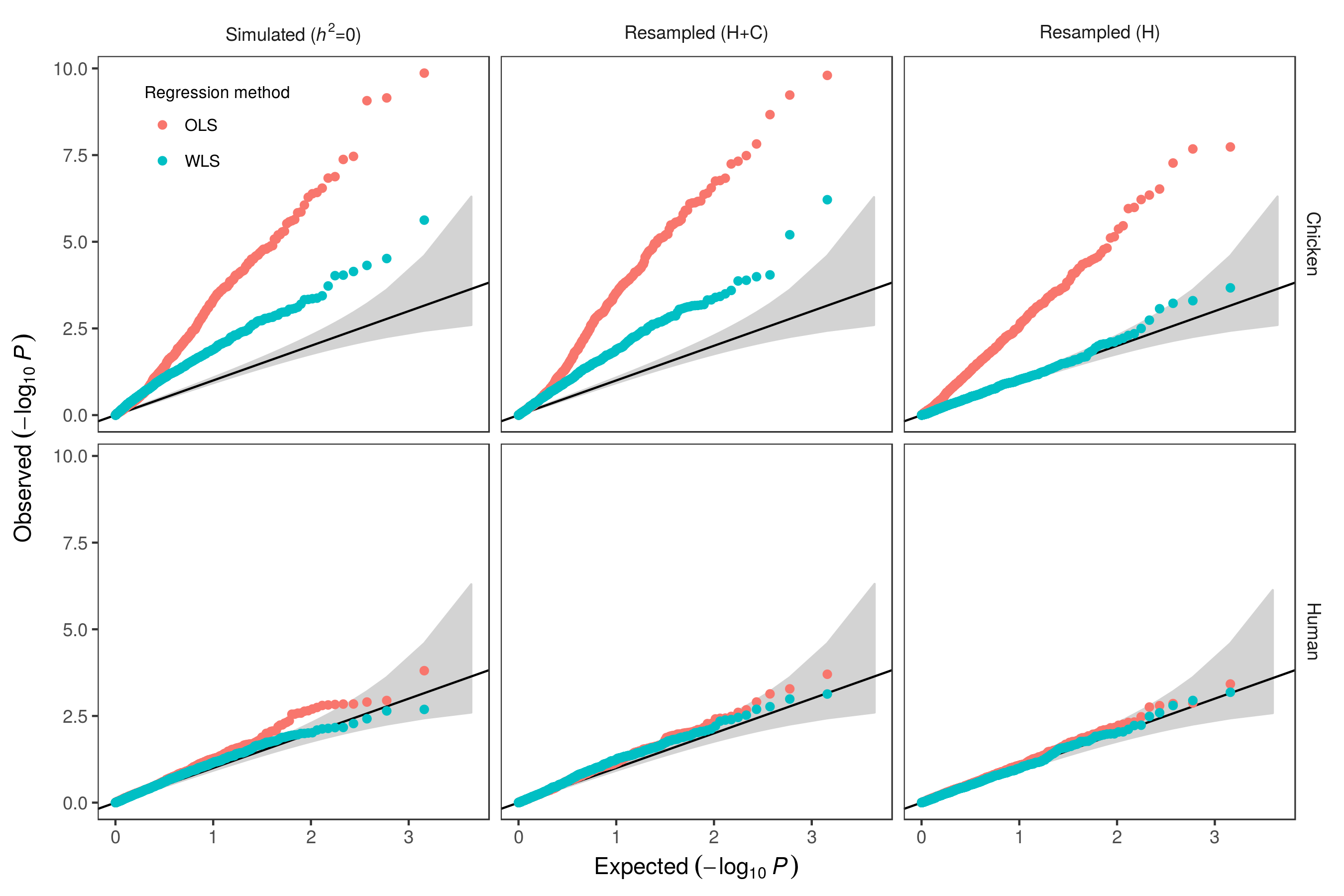
**Supplementary Figure S1.** Ordinary least squares (OLS) regression versus weighted least squares regression (WLS) with heteroscedasticity and censoring. Shows QQ-plot of expected (uniform distributions between 0 and 1) versus observed -log_10_ *P* values from regression between *h^2^_c_* and chromosome size*.* Data are either simulated under the null hypothesis of no association between phenotype and genotype (*h^2^*=0; based on the simulated data in Fig. 1, main text; n=1000) or resampled with heteroscedasticity, with (H+C) or without (H) censoring (see main text for details). Grey area shows 95% confidence interval and solid black line indicates 1:1 line.

**
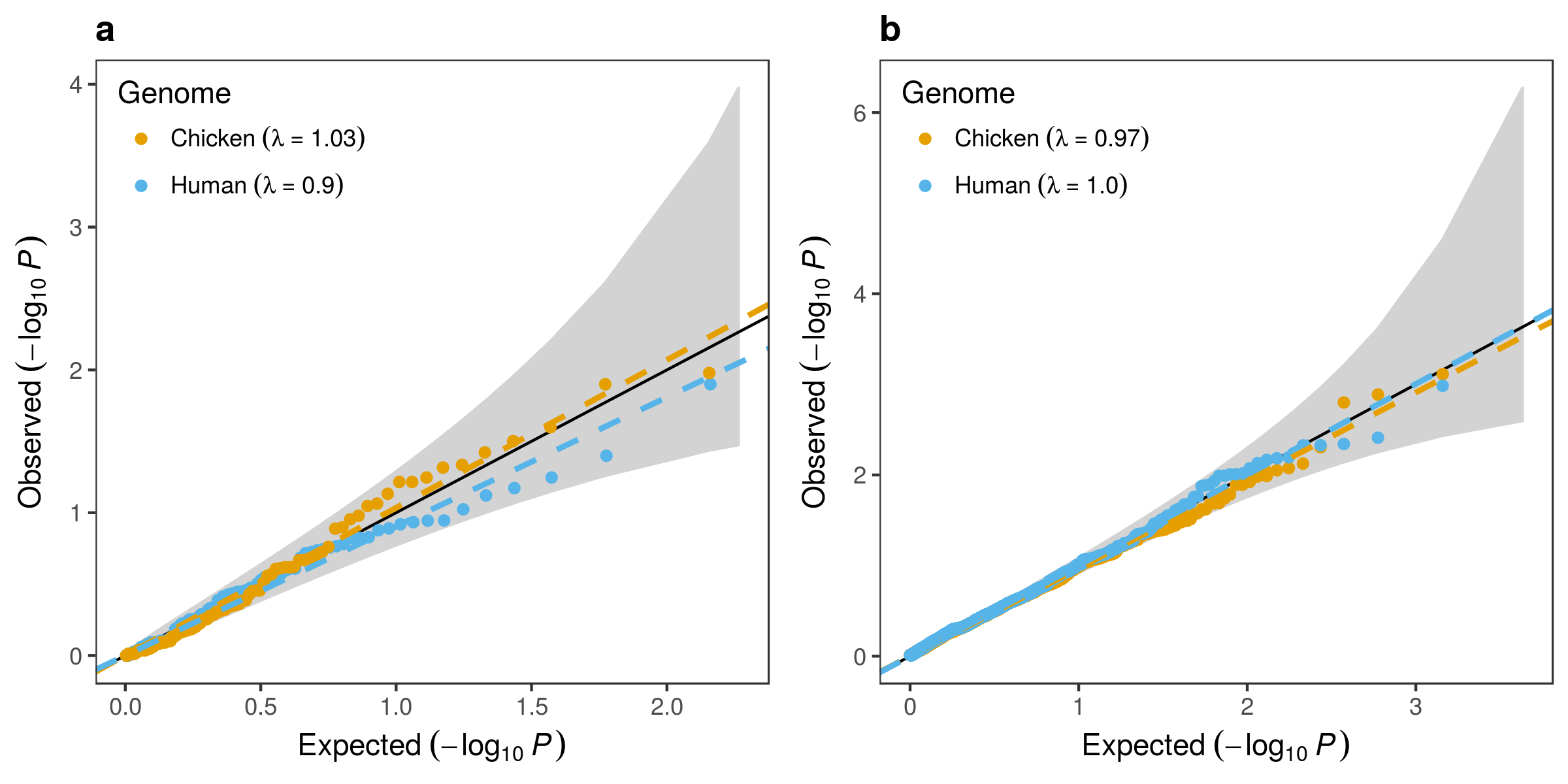
**

**Supplementary Figure S2.** Correction of *P* value inflation under the null hypothesis using permutation or resampling with heteroscedasticity and censoring. Shows QQ-plot of expected (uniform distributions between 0 and 1) versus observed -log_10_ *P* values from regression between *h^2^_c_ and* chromosome size when *h^2^*=0*.* (**a**) Null distribution of *P* values is based on permutation of phenotypic values prior to chromosome partitioning analyses (100 data sets). In (**b**) null distribution for *P* values is based on resampling of original *h^2^_c_* estimates for the regression from a normal distribution with mean equal to zero and *sd* equal to *SE_h_* (1000 data sets) and censoring as in the original data. *P* value inflation (λ, indicated in figure) is the slope for the regression line (dashed). Grey area shows 95% confidence interval and solid black line shows 1:1 line.


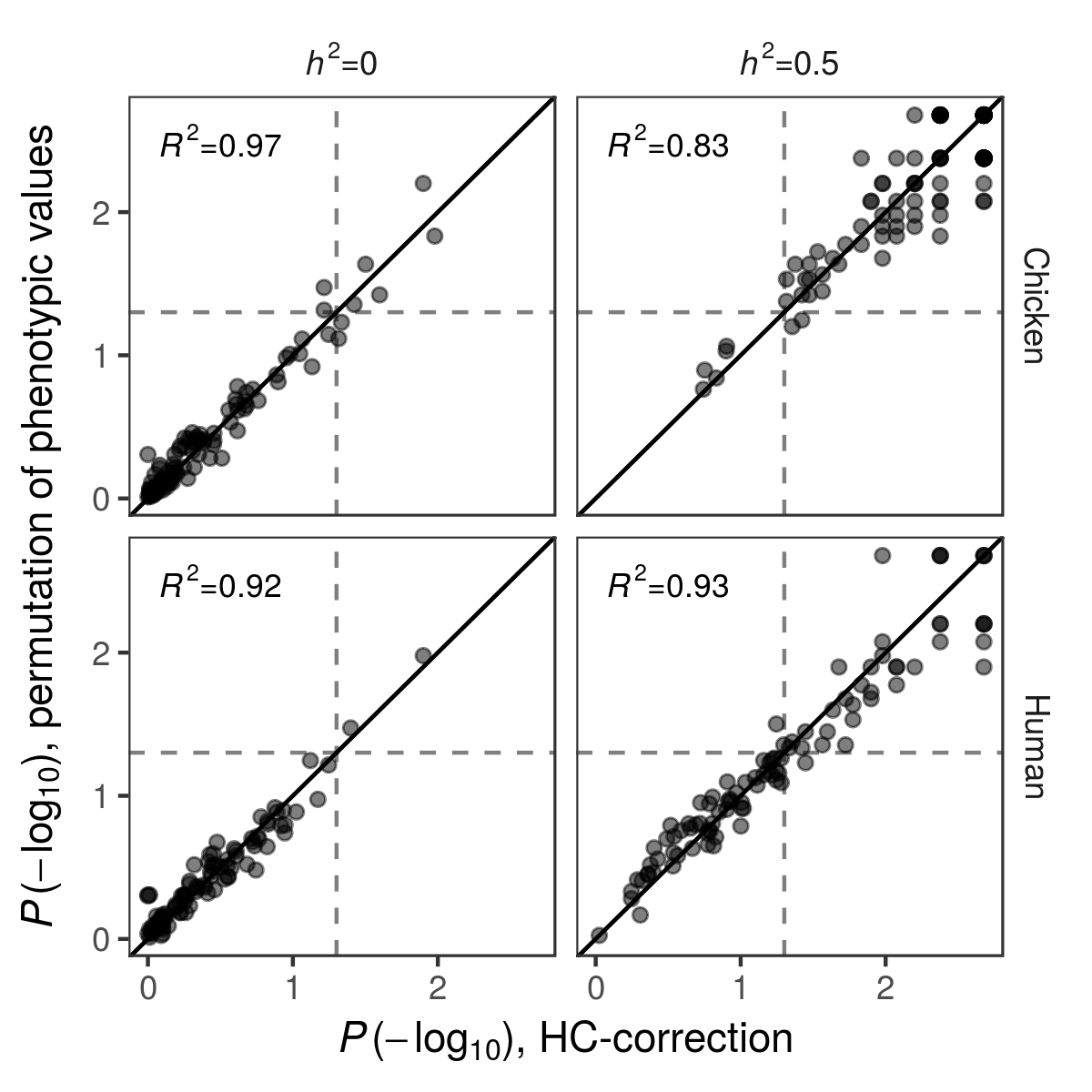
**Supplementary Figure S3.** *P* value correction using null distribution from permutation or resampling with heteroscedasticity and censoring. A null-distribution for *P* values from OLS regression was generated by either permuting phenotypic values among individuals prior to chromosome partitioning or by resampling each data point (with heteroscedasticity and censoring) in the regression between *h^2^_c_* and chromosome size. Based on 100 simulated data sets for each genome (chicken or human) with *h^2^=0* or *h^2^=*0.5 (polygenic trait inheritance). The number of replicates was limited to 475 (adaptive resampling), setting the upper limit for –log_10_ *P* values to 2.67 (see main text for details). Adjusted R^2^ are indicated in the figure. -log_10_ *P* values from permutation and HC-resampling do not differ significantly from each other (paired *t*-tests; all *P* > 0.05). See Methods for additional simulation parameters. Horizontal and vertical dashed lines in indicate significance level at α=0.05; in unbiased tests ~5% of values under the null hypothesis are expected to significant.


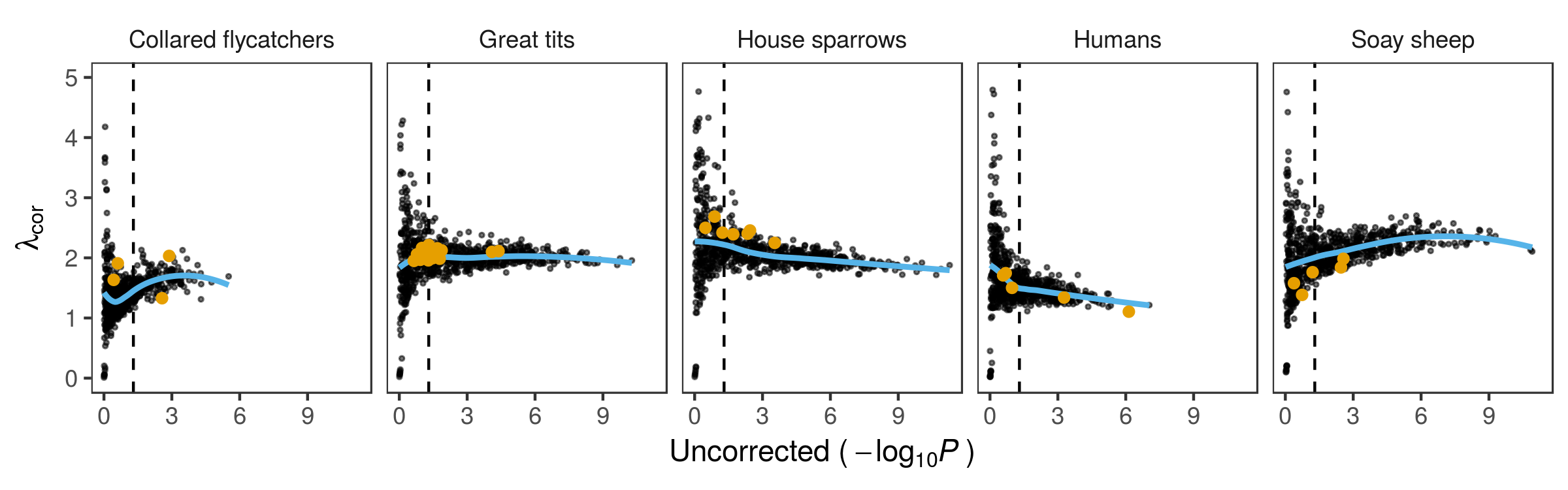
**Supplementary Figure S4.** The ratio between HC-corrected *P* values and uncorrected *P* values (λ_cor_) depends on the strength of correlation between *h^2^_c_* and chromosome size. λ_cor_ is shown as a function of uncorrected -log_10_ *P* value from an OLS regression between *h^2^_c_* and chromosome size. Black data points are based on simulate data and yellow points represent empirical data (figure based on data from Fig. 4, main text and Supporting Table S1). Blue line represents a loess regression line. Dashed vertical line represents significance level at α=0.05. The trends between simulated and empirical data (within species) are much more similar within species than between species. Black dots represent simulated data and yellow dots represent empirical data. λ_cor_ represent point estimate of *P* value inflation (see main text), which is not constant across the range of uncorrected *P* values, but always >1 (indicating that *P* value inflation) for tests where the uncorrected P value is significant (right of the vertical dashed line).


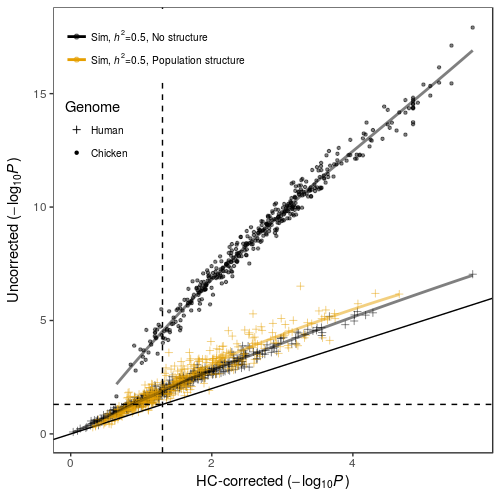


**Supplementary figure S5.** Relationship between uncorrected and HC-corrected *P* in simulated data with population structure. In data simulated with and without population structure are shown in yellow and black, respectively. Data from simulations based on the chicken genome (filled circles) is shown as a contrast to simulations based on the human genome (‘+’). Note that it was not possible to perform these analyses on data sets with population structure with the chicken genome (due to convergence issues); for details on simulation parameters, see main text. Horizontal and vertical dashed lines indicate significance level at α=0.05, such that data points in the upper left quadrant represent false positives, if not accounting for heteroscedasticity and censoring.

**Supplementary table S1.** Uncorrected (OLS) and HC-corrected (HC) *P* values from published chromosome partitioning analyses.

Genome Trait *P* (OLS) *P* (HC) λ_cor_

Humans Height 7.29E-07* 2.80E-06* 1.11

Humans Weight 2.01E-01 3.99E-01 1.75

Humans BMI 1.06E-01 2.24E-01 1.50

Humans vWF 2.62E-01 4.57E-01 1.71

Humans QTi 5.33E-04* 3.61E-03* 1.34

House sparrows Tarsus length 3.57E-03 1.01E-01 2.45

House sparrows Wing length 3.32E-01 6.44E-01 2.50

House sparrows Mass **1.97E-02*** 1.94E-01 2.39

House sparrows Bill depth 5.89E-02 3.10E-01 2.42

House sparrows Bill length 2.90E-04* 2.67E-02* 2.25

House sparrows Total badge 1.32E-01 4.71E-01 2.69

House sparrows Visible badge **4.54E-03*** 1.06E-01 2.40

Collared flycatchers Tarsus length 2.70E-03* 1.17E-02* 1.33

Collared flycatchers Wing length 1.31E-03* 3.82E-02* 2.03

Collared flycatchers Mass 3.71E-01 5.45E-01 1.63

Collared flycatchers White patch 2.45E-01 4.78E-01 1.91

Great tits Clutch size NL **9.92E-02*** 3.44E-01 2.16

Great tits Clutch size UK **2.10E-02*** 1.67E-01 2.16

Great tits Egg mass UK 8.01E-05* 1.12E-02* 2.10

Great tits Fledgling weight (of offspring) NL **4.99E-02*** 2.58E-01 2.21

Great tits Fledgling weight (of offspring) UK 1.65E-01 4.17E-01 2.06

Great tits Adult weight NL 5.21E-02 2.21E-01 1.96

Great tits Adult weight UK **1.34E-02*** 1.32E-01 2.12

Great tits Fledgling weight (of individual) NL **4.10E-02*** 2.23E-01 2.13

Great tits Fledgling weight (of individual) UK 5.70E-02 2.52E-01 2.08

Great tits Tarsus length NL 4.11E-05* 8.37E-03* 2.11

Great tits Tarsus length UK 1.18E-01 3.37E-01 1.97

Great tits Wing length NL 2.20E-01 4.60E-01 1.95

Great tits Wing length UK **1.64E-02*** 1.26E-01 1.99

Great tits Exploratory behaviour NL **3.37E-02*** 1.83E-01 1.99

Great tits Exploratory behaviour UK 2.28E-01 4.69E-01 1.95

Soay sheep Foreleg 1.82E-01 2.92E-01 1.38

Soay sheep Hindleg 6.23E-02 2.07E-01 1.76

Soay sheep Metacarpal 4.16E-01 5.73E-01 1.58

Soay sheep Weight **2.71E-03*** 5.09E-02 1.98

Soay sheep Jaw 3.47E-03* 4.61E-02* 1.84

λ_cor_ is the ratio between uncorrected and HC-corrected *P* values (at the -log_10_-scale), which indicates the magnitude of *P* value inflation. * Indicates significant results at α=0.05, and *P* values in bold represent false positives (a significant test before, but not after HC-correction).
